# Supplementary material for: Generation of axenic Aedes aegypti demonstrate live bacteria are not required for mosquito development
Source: Nat Commun. 2018 Oct 26;9:4464. doi: 10.1038/s41467-018-07014-2 (PMC6203775; doi:10.1038/s41467-018-07014-2)
Supplement: Supplementary file 1 — Supplementary Information [file 41467_2018_7014_MOESM1_ESM.pdf]

Supplementary Figure 1

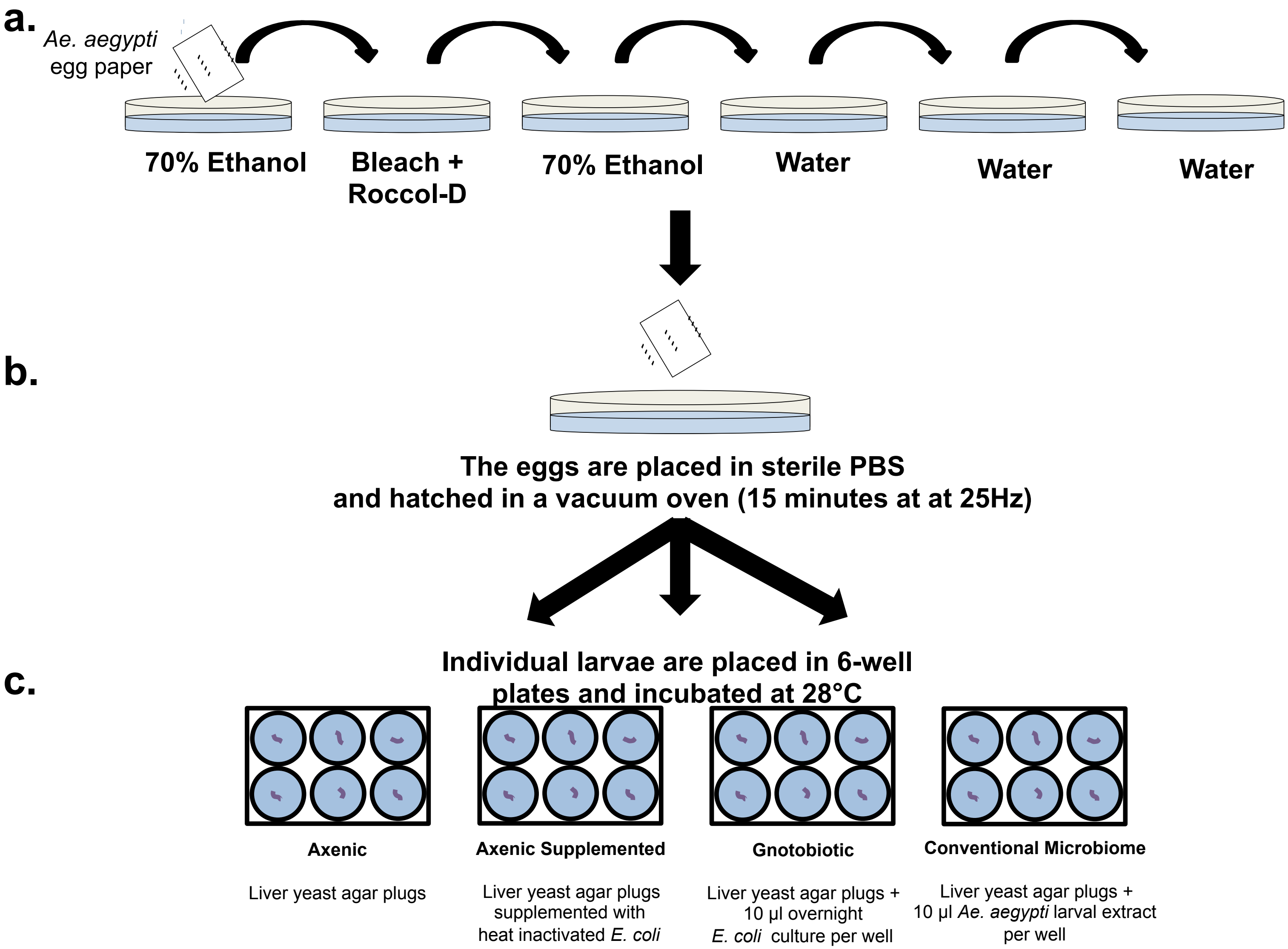

Supplementary Figure 1. Schematic diagram of egg sterilization. *Ae. aegypti* eggs were collected from colony reared mosquitoes and surface sterilized as depicted. (a) Eggs were serially washed for 5 minutes in each solution. (b) Surface sterilized eggs were transferred to a Petri dish containing sterile PBS (c) After hatching the eggs in a vacuum oven, individual larvae were transferred from the Petri dishes to individual wells of a six well plate for development assays.

Supplementary Figure 2

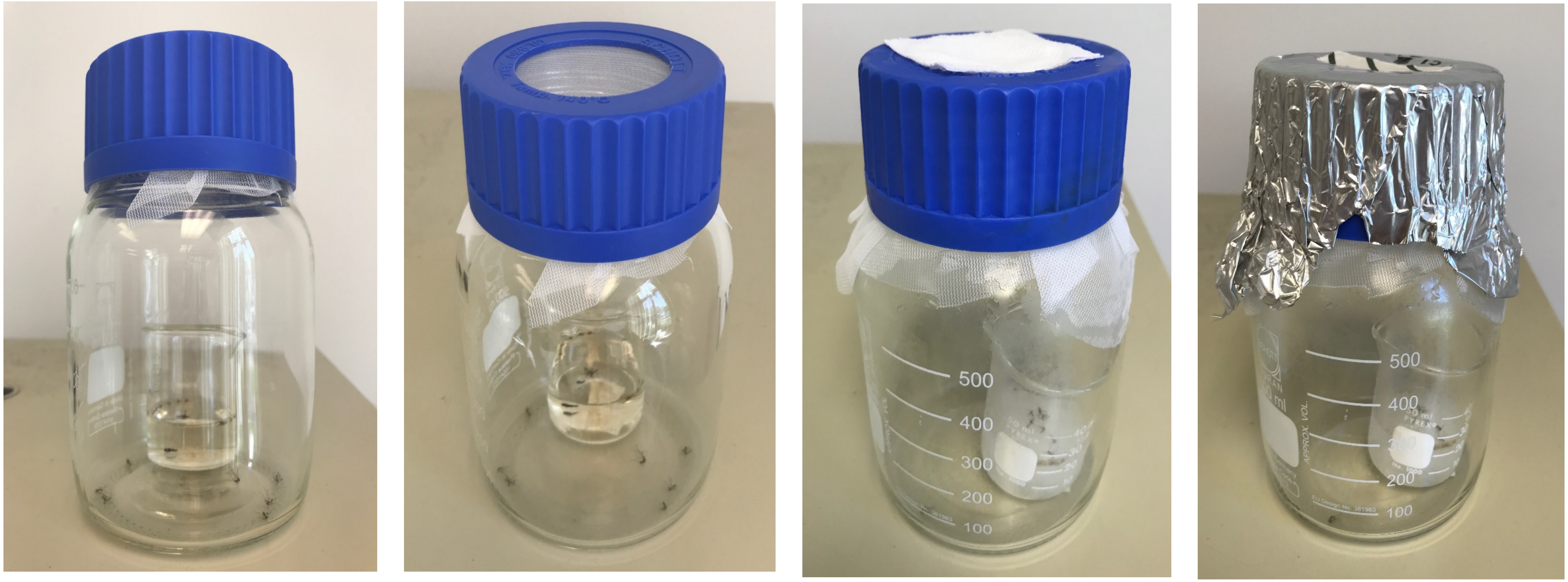

Supplementary Figure 2. Mosquito emergence jars. The jar consisted of a 500 ml Duran wide mouth glass bottle with a 50 ml glass beaker placed inside (A). The top of the jar was covered with cloth mesh and the apparatus was sealed with a lid, in which a 5 cm diameter hole was cut into the center (B). A cotton pad was placed on top of the mesh (C) and the apparatus was sealed with aluminum foil (D) and autoclaved. Upon pupation, individual mosquitoes were transferred to the 50 ml glass beaker filled with 20 ml sterile PBS. Mosquito feeding was performed in a laminar flow hood by pipetting 1 ml of a filter sterilized 10% sucrose solution onto the cotton pad.

Supplementary Figure 3

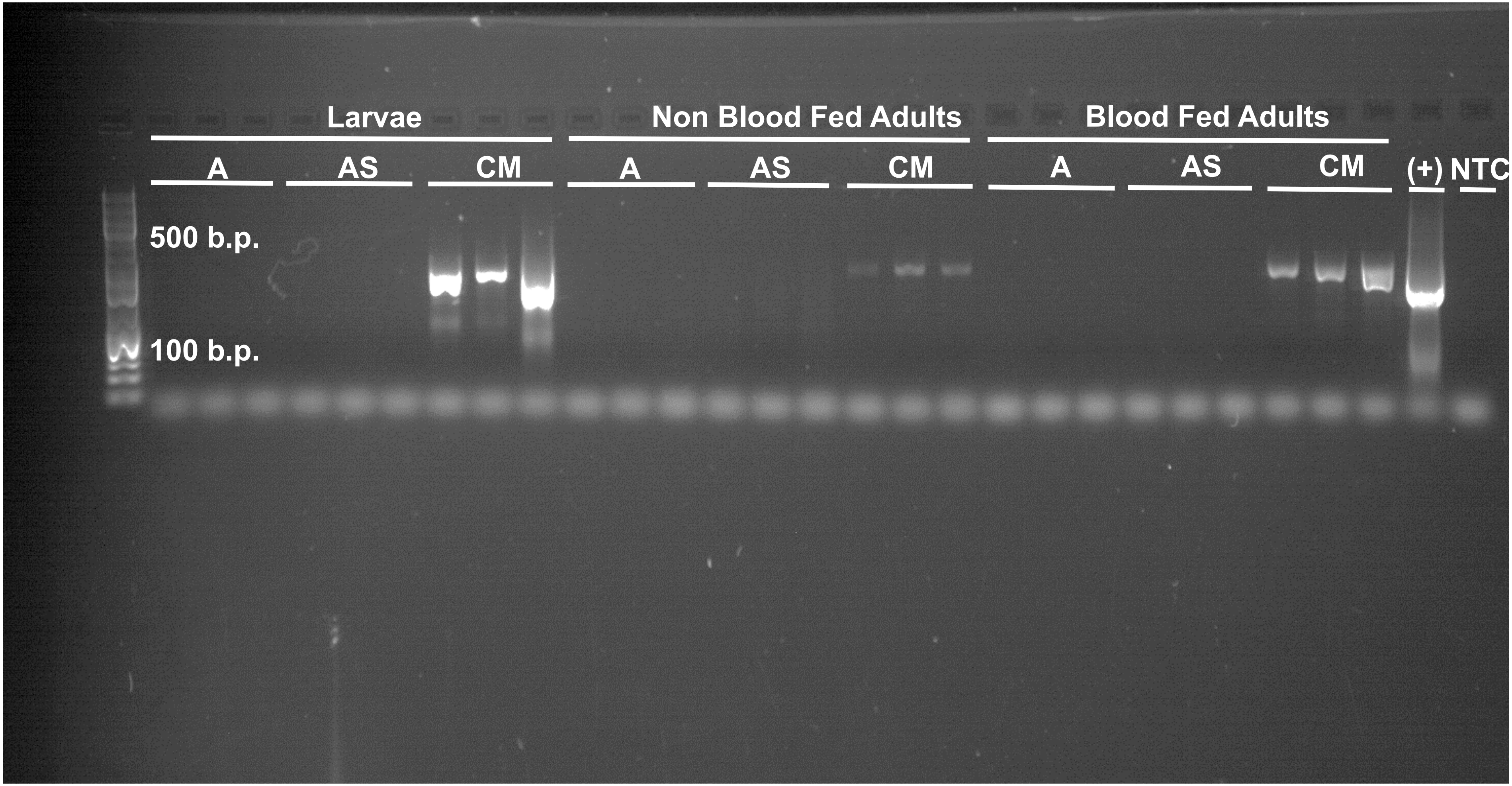

Supplementary Figure 3. PCR detection of bacterial DNA (uncropped gel). Total DNA was extracted from axenic (A), axenic supplemented with heat killed *E. coli* (AS), and colony microbiome (CM) mosquitoes at three different developmental stages (larva, non-blood fed adult, and blood fed adult). DNA from three individuals from each group was used as a template for amplification of bacterial 16S rRNA genes. A and AS mosquitoes show no visible PCR products across all developmental stages, whereas amplification products were identified in the CM mosquitoes. A positive control (+) containing amplified *E. coli*-- -K-12 DNA and a non-template control (NTC) are also included on the gel. Amplification consisted of 30 cycles and an annealing temperature of 55°C (see methods).
